# 

**Published:** 1898-01

**Authors:** 


### LIST OF CONTRIBUTORS TO VOLUME XIX.

Allan, George S., D.D.S.
Bauchwitz, Max.
Belcher, Harry L., D.D.S.
Black, G. V., M.D., D.D.S.
Bonwill, W. G. A., D.D.S.
Brackett, Charles A., D.M.D.
Bryan, L. C., D.D.S.
Bull, Charles Stedman, A.M., M.D.
Burchard, Henry H., M.D., D.D.S.
Chance, George H., M.D., D.D.S.
Clapp, Dwight M., D.M.D.
Codman, J. T., D.M.D.
Davenport, S. E., D.D.S.
Davenport, W. S.
Dunn, William, D.D.S.
Fillebrown, Dr. Thomas.
Gartrell, J. H.
Howe, J. Morgan, M.D.S., M.D.
Hubbard, Dwight L., M.D.
Inglis, Otto E., D.D.S.
Jack, L. Foster, M.D., D.D.S.
Jack, Louis, D.D.S.
Kimball, Charles O., M.D.
Lord, Benjamin, D.D.S.
Luckie, S. Blair, D.D.S.
Lund, F. B., M.D.
Mills, Dr. G. Alden.
Musson, Dr. Emma E.
Nones, Robert H., D.D.S.
Owen, John, M.B.
Peirce, C. N., D.D.S.
Perry, Safford G., D.D.S.
Potter, William H., D.M.D.
Rogers, John, M.D.
Rollins, William.
Scott, J. Alfred, M.A., M.D., F.R.C.S.I.
Smith, B. Holly, M.D., D.D.S.
Taft, Charles H., A.B., D.M.D.
Talbot, Eugene S., M.D., D.D.S.
Trueman, William H., D.D.S.
Underwood, Arthur S., L.D.S. M.R.C.S.
Warren, George W., D.D.S.
Whittles, Dencer, L.D.S.
Worcester, J. W., M.D.
York, E. Lawley, D.D.S.

